# Direct Immunofluorescence Intensity as a Supportive Marker of Clinical Severity in Pemphigus Vulgaris

**DOI:** 10.7759/cureus.112255

**Published:** 2026-07-08

**Authors:** Harshita Reddy Bondugula, Samhitha Mallavalli, Tejaswi Kothapally, Ashok Babu Venna, Sriteja Vemulapalli, Manmohan G, Muralidhar Chinnapaka

**Affiliations:** 1 Department of Dermatology, Apollo Institute of Medical Sciences and Research, Hyderabad, IND; 2 Deparment of Dermatology, Gandhi Medical College, Secunderabad, IND; 3 Department of Dermatology, Patnam Mahender Reddy Institute of Medical Sciences, Hyderabad, IND; 4 Department of Dermatology, Skin Veda Clinics, Hyderabad, IND; 5 Department of Pharmacology, Government Medical College and Hospital Maheshwaram, Hyderabad, IND

**Keywords:** autoimmune blistering disease, c3, direct immunofluorescence, igg, pemphigus disease area index, pemphigus vulgaris

## Abstract

Background

Pemphigus vulgaris is an autoimmune blistering disorder in which autoantibody-mediated loss of keratinocyte adhesion leads to mucosal and cutaneous erosions. Direct immunofluorescence (DIF) is routinely used to confirm the diagnosis by demonstrating intercellular IgG deposition, with or without C3, in a fishnet or chicken-wire pattern. Although DIF is primarily a diagnostic test, its staining intensity and immune-reactant pattern may reflect the burden of tissue-bound immune activity. This study assessed the association between DIF findings and clinical severity in patients with pemphigus vulgaris.

Materials and methods

This prospective observational study included 60 patients with clinically and histopathologically confirmed pemphigus vulgaris. Clinical severity was assessed at presentation using the pemphigus disease area index (PDAI). DIF was performed on perilesional skin or mucosal biopsy specimens using fluorescein-labelled antisera against IgG, IgA, IgM, C3, and fibrinogen. The type, pattern, and intensity of immune deposits were recorded. DIF intensity was graded semi-quantitatively as 0, 1+, 2+, or 3+. The relationship between DIF intensity and PDAI score was analyzed using Spearman’s rank correlation coefficient. Associations between DIF findings and severity categories were assessed using the chi-square test or Fisher’s exact test, as appropriate.

Results

The mean age of the patients was 42.6 ± 13.8 years. There were 34 (56.7%) females and 26 (43.3%) males. Mucocutaneous involvement was the most frequent clinical pattern, observed in 38 (63.3%) patients. Based on PDAI grading, 18 (30.0%) patients had mild disease, 26 (43.3%) had moderate disease, and 16 (26.7%) had severe disease. Intercellular IgG deposition was detected in 58 (96.7%) patients, while combined IgG and C3 deposition was observed in 42 (70.0%) patients. Strong DIF staining was seen more often in patients with severe disease. The mean PDAI score increased across DIF grades, from 11.8 ± 4.3 in patients with weak staining to 45.6 ± 12.1 in those with strong staining. DIF intensity showed a moderate positive correlation with PDAI score (Spearman’s rho = 0.62, p < 0.001). Combined IgG and C3 deposition was also significantly associated with moderate-to-severe disease (p = 0.004).

Conclusion

DIF intensity showed a significant positive association with clinical severity in pemphigus vulgaris. Strong intercellular IgG staining and combined IgG-C3 deposition were more frequent among patients with higher PDAI scores. These findings suggest that semi-quantitative DIF reporting may provide useful supportive information at baseline when interpreted together with clinical severity scoring. However, as this study demonstrates association only, DIF intensity should not be considered an independent predictive or prognostic marker without longitudinal validation.

## Introduction

Pemphigus vulgaris is a potentially serious autoimmune blistering disorder involving the skin and mucous membranes. The disease is primarily mediated by autoantibodies directed against desmoglein 3, and in many mucocutaneous cases, desmoglein 1 is also involved. These desmosomal cadherins are essential for adhesion between keratinocytes. Loss of this adhesion leads to suprabasal acantholysis, producing fragile blisters and erosions that are characteristic of the disease [1,2].

Clinically, pemphigus vulgaris often begins with painful oral erosions, which may precede cutaneous lesions by several weeks or months. Skin involvement usually appears as flaccid bullae that rupture easily, leaving raw erosions and crusted areas. Because the disease follows a chronic and relapsing course, patients may experience considerable morbidity due to pain, secondary infection, impaired oral intake, fluid loss, nutritional difficulty, and adverse effects related to prolonged immunosuppressive therapy [3].

The diagnosis of pemphigus vulgaris requires clinicopathological correlation. Histopathology typically shows suprabasal clefting with acantholytic keratinocytes. Direct immunofluorescence (DIF) remains a key diagnostic investigation and usually demonstrates intercellular IgG deposition in a fishnet or chicken-wire pattern. Complement C3 may also be detected in some cases, particularly when the disease is clinically active. Unlike serological assays, DIF demonstrates tissue-bound immune deposits at the site of disease activity, which makes it a useful and dependable test in routine dermatopathology practice [4,5].

Assessment of disease severity is important because pemphigus vulgaris shows wide variation in extent, activity, and clinical burden. Patients with limited oral disease may require a different treatment approach from those with extensive mucocutaneous involvement. The pemphigus disease area index (PDAI) is a validated scoring system that separately assesses skin, scalp, and mucosal activity. It is useful for documenting baseline severity and monitoring disease course during follow-up. The Autoimmune Bullous Skin Disorder Intensity Score is another validated instrument used in the clinical assessment of autoimmune blistering diseases [6-9].

Most available literature has examined the relationship between circulating anti-desmoglein 1 and anti-desmoglein 3 antibody levels and clinical severity in pemphigus vulgaris [10-13]. These serological markers are valuable because they provide quantitative information and may help in monitoring disease activity. However, enzyme-linked immunosorbent assay (ELISA) testing may not be routinely available in all clinical settings. In contrast, DIF is more commonly used as part of the diagnostic workup in many dermatology and pathology services. Despite this, the possible clinical relevance of semi-quantitative DIF findings, especially staining intensity and associated C3 deposition, has received comparatively less attention [14-17].

Although DIF is primarily a diagnostic tool, its staining intensity and immune-reactant pattern may offer additional supportive information at the time of initial evaluation. Strong intercellular IgG staining and combined IgG-C3 deposition may indicate a higher burden of tissue-bound immune activity. At the same time, DIF intensity should be interpreted cautiously because it can be influenced by several factors, including disease duration, biopsy site, prior treatment, specimen transport, tissue preservation, microscope settings, and observer interpretation. DIF positivity may also persist despite clinical improvement; therefore, it cannot be considered an independent prognostic or predictive marker without longitudinal evidence [5,9].

The novelty of the present study lies in evaluating whether tissue-bound immune deposits on DIF, particularly the intensity of intercellular IgG staining and the presence of C3 deposition, are associated with PDAI-based clinical severity at presentation. This question is clinically relevant in settings where serum anti-desmoglein antibody estimation is not consistently available, but DIF is routinely performed for diagnosis.

The present study was therefore conducted to assess the association between DIF findings and clinical severity in patients with pemphigus vulgaris. The study evaluated the type, pattern, and intensity of immune deposits and examined whether stronger IgG staining and combined IgG-C3 deposition were associated with higher PDAI scores. The aim was not to establish DIF intensity as a prognostic marker but to determine whether it can provide supportive information when interpreted along with standard clinical severity scoring.

## Materials and methods

Study design

This was a prospective observational study that included patients with confirmed pemphigus vulgaris. The study was planned to assess whether DIF findings, especially intercellular IgG staining intensity and C3 deposition, were associated with clinical severity at the time of presentation. Since the design was observational and baseline-based, the study evaluated association only and was not intended to determine the predictive or prognostic value of DIF intensity.

Study setting

The study was conducted at the Department of Dermatology, Apollo Institute of Medical Sciences and Research, Hyderabad, Telangana, India, in collaboration with the Department of Pathology. Patients were clinically evaluated in the Department of Dermatology, and histopathological examination and DIF testing were performed with pathology support. Standard institutional protocols were followed for biopsy collection, specimen transport, processing, and interpretation.

Study duration

The study was carried out over a two-year period from May 2024 to April 2026. During this period, patients presenting with clinical features suggestive of pemphigus vulgaris were screened, and those fulfilling the eligibility criteria were enrolled after obtaining written informed consent. Clinical severity assessment, biopsy collection, and DIF testing were performed at the time of initial evaluation before starting or modifying systemic treatment whenever possible.

Study population

The study population consisted of patients attending the dermatology outpatient department or admitted with clinical features suggestive of pemphigus vulgaris. Each patient underwent detailed clinical examination, histopathological confirmation, and DIF testing. Only cases with adequate clinical records, confirmatory histopathology, and interpretable DIF findings from perilesional skin or mucosal biopsy were included in the final analysis. Disease duration, site of involvement, prior treatment history, and biopsy site were recorded because these factors could potentially influence DIF staining intensity.

Inclusion criteria

Patients aged 18 years and above were included if they had clinical features consistent with pemphigus vulgaris and histopathological evidence of suprabasal acantholysis. Cases were included only when DIF findings were available from an adequate perilesional skin or mucosal biopsy specimen. Patients were enrolled after providing written informed consent.

Exclusion criteria

Patients who had received systemic corticosteroids or other immunosuppressive therapy for more than two weeks before biopsy were excluded to reduce the possible effect of prior treatment on DIF staining intensity. Patients with other autoimmune blistering diseases, including pemphigus foliaceus, paraneoplastic pemphigus, bullous pemphigoid, mucous membrane pemphigoid, dermatitis herpetiformis, and linear IgA disease, were excluded. Cases with suspected drug-induced blistering disorders, inadequate biopsy tissue, delayed or improper specimen transport, poorly preserved samples, or non-interpretable DIF slides were also excluded.

Sample size and its calculation

The sample size was calculated for assessing the correlation between DIF intensity and clinical severity measured by PDAI. Since DIF intensity was graded as an ordinal variable and PDAI was recorded as a clinical severity score, a sample size formula for correlation analysis using Fisher’s z transformation was applied:

n = [(Zα + Zβ) / C]² + 3

where:

C = 0.5 × ln[(1 + r) / (1 − r)]

In the above formula, n represents the required sample size, Zα represents the standard normal deviate for the selected level of significance, Zβ represents the standard normal deviate for study power, and r represents the expected correlation coefficient. Assuming an anticipated moderate correlation coefficient of 0.40 between DIF intensity and PDAI score, with 80% power and 5% level of significance, the minimum sample size required was approximately 47 patients. After allowing for incomplete clinical data, inadequate biopsy samples, and non-interpretable DIF findings, 60 patients were included. PDAI was selected because it is a validated severity assessment tool for pemphigus [6].

Clinical assessment

A detailed history was obtained from each patient, including age, sex, duration of disease, first site of lesion, mucosal symptoms, extent of skin involvement, pain, dysphagia, previous treatment, and recurrence. All patients underwent general physical examination followed by complete dermatological examination. Disease severity was assessed using the PDAI. Based on the PDAI score, patients were classified as having mild disease when the score was less than 15, moderate disease when the score was 15 to 45, and severe disease when the score was more than 45. The PDAI score was used for comparison with DIF intensity and C3 deposition.

Biopsy procedure

Wherever feasible, two biopsy specimens were obtained from each patient. One biopsy was taken from a representative lesional area for routine histopathological examination and fixed in 10% neutral buffered formalin. The second biopsy was taken from perilesional normal-appearing skin or mucosa, preferably within 1 cm of an active lesion, for DIF. The biopsy site was documented as skin or mucosa because site-related variation may influence DIF staining. The DIF specimen was transported in Michel’s medium or normal saline according to laboratory protocol and processed as early as possible to preserve immune-reactant quality.

Direct immunofluorescence

For DIF testing, frozen tissue sections were prepared and incubated with fluorescein isothiocyanate-labelled antisera against IgG, IgA, IgM, C3, and fibrinogen. Slides were examined under a fluorescence microscope. The site of deposition, pattern of fluorescence, type of immune reactant, staining intensity, and presence or absence of complement C3 were recorded. Intercellular epidermal or epithelial staining in a fishnet or chicken-wire pattern was considered characteristic of pemphigus vulgaris. DIF findings were interpreted along with the clinical and histopathological diagnosis.

DIF intensity grading

DIF intensity was assessed using a semi-quantitative grading system. A negative result was recorded as 0. Faint but definite intercellular fluorescence was graded as 1+ weak. Clearly visible intercellular fluorescence of intermediate brightness was graded as 2+ moderate. Bright and strong intercellular fluorescence in the typical fishnet or chicken-wire pattern was graded as 3+ strong. These grades were compared with the PDAI score and clinical severity category.

To improve clarity and reproducibility, representative pictorial examples of weak, moderate, and strong DIF staining were included in the revised figure section. The intensity grading was based on visual assessment under fluorescence microscopy. However, as this was a semi-quantitative method, possible observer variation was acknowledged. Formal interobserver reliability testing was not performed, and this has been added as a limitation of the study.

Assessment of potential confounders

Possible factors that could affect DIF intensity were considered during study design and analysis. Disease duration, prior systemic treatment, biopsy site, specimen handling, and tissue preservation were recorded wherever available. Patients with more than two weeks of systemic corticosteroid or immunosuppressive treatment before biopsy were excluded to reduce treatment-related attenuation of staining. Biopsy site was documented as skin or mucosa, and its possible influence on DIF intensity was considered during interpretation. Since the study sample was modest, multivariable adjustment for all potential confounders was limited, and this has been acknowledged in the revised manuscript.

Statistical analysis

Data were entered into Microsoft Excel (Microsoft Corporation, Redmond, WA) and analyzed using SPSS Version 26.0 (IBM Corp., Armonk, NY). Continuous variables were expressed as mean ± standard deviation. Categorical variables were presented as number and percentage, n (%). Associations between categorical variables were analyzed using the chi-square test or Fisher’s exact test, as appropriate. The association between DIF intensity grade and clinical severity category was assessed using the chi-square test, and the chi-square value, degrees of freedom, and p-value were reported. The relationship between DIF intensity and PDAI score was analyzed using Spearman’s rank correlation coefficient because DIF intensity was an ordinal variable. A p-value of less than 0.05 was considered statistically significant, while p<0.001 was considered highly significant.

Ethical considerations

The study was conducted after obtaining approval from the Institutional Ethics Committee of Gandhi Medical College, Secunderabad, Telangana, India, in memorandum of understanding (MOU) with Apollo Institute of Medical Sciences and Research. The approval number was GMC/IEC/DVL/2024-12. Written informed consent was obtained from all participants before enrolment. The study was conducted in accordance with the ethical principles of the Declaration of Helsinki. Patient confidentiality was maintained throughout the study, and personal identifiers were not disclosed. Biopsy procedures were performed under aseptic precautions, and all patients received standard treatment according to disease severity.

## Results

Demographic profile

A total of 60 patients with pemphigus vulgaris were included in the study. The age of the patients ranged from 19 to 72 years, with a mean age of 42.6 ± 13.8 years. There were 34 (56.7%) females and 26 (43.3%) males, giving a female-to-male ratio of 1.3:1 (Table 1).

**Table 1:** Demographic profile of study participants Data are presented as number and percentage, n (%), for categorical variables and mean ± standard deviation (SD) for age.

Variable	Number of patients	Percentage
Total cases	60	100.0
Age <30 years	11	18.3
Age 31 to 50 years	32	53.3
Age >50 years	17	28.4
Male	26	43.3
Female	34	56.7
Mean age	42.6 ± 13.8 years	-

Clinical pattern

Oral mucosal involvement was the most frequent clinical feature and was observed in 52 (86.7%) patients. Cutaneous erosions were seen in 46 (76.7%) patients, flaccid bullae in 32 (53.3%) patients, and crusted lesions in 39 (65.0%) patients. Dysphagia or painful swallowing was reported by 18 (30.0%) patients, while genital mucosal involvement was present in 7 (11.7%) patients. Nikolsky sign was positive in 46 (76.7%) patients (Table 2).

**Table 2:** Clinical presentation of pemphigus vulgaris Data are presented as number and percentage, n (%). Percentages were calculated using the total study population as the denominator, n=60.

Clinical feature	Number of patients	Percentage
Oral erosions	52	86.7
Cutaneous erosions	46	76.7
Flaccid bullae	32	53.3
Crusting lesions	39	65.0
Dysphagia or painful swallowing	18	30.0
Genital mucosal involvement	7	11.7
Positive Nikolsky sign	46	76.7

Clinical severity based on PDAI

Based on PDAI grading, 18 (30.0%) patients had mild disease, 26 (43.3%) patients had moderate disease, and 16 (26.7%) patients had severe disease. The mean PDAI score was 31.4 ± 17.6 (Table 3).

**Table 3:** Distribution of disease severity based on PDAI Data are presented as number and percentage, n (%). Disease severity was categorized according to the PDAI score. PDAI, pemphigus disease area index

Disease severity	PDAI range	Number of patients	Percentage
Mild	<15	18	30.0
Moderate	15 to 45	26	43.3
Severe	>45	16	26.7

The mean PDAI score was 31.4 ± 17.6, indicating that a considerable proportion of patients had clinically significant disease at presentation. Based on PDAI grading, 18 patients were classified as having mild disease, 26 patients had moderate disease, and 16 patients had severe disease. Thus, moderate disease was the most common severity category in this study, followed by mild and severe disease groups.

DIF findings

DIF was positive in 58 (96.7%) patients and negative in two (3.3%) patients. Intercellular IgG deposition was observed in 58 (96.7%) patients, while intercellular C3 deposition was seen in 42 (70.0%) patients. IgA alone was observed in 16 (26.7%) patients, weak IgM in 4 (6.7%) patients, and weak IgA in 3 (5.0%) patients. Combined IgG and C3 deposition was present in 42 (70.0%) patients (Table 4).

**Table 4:** Pattern of immune deposits on DIF Data are presented as number and percentage, n (%). Percentages were calculated using the total study population, n=60. DIF, direct immunofluorescence

DIF finding	Number of patients	Percentage
Intercellular IgG	58	96.7
Intercellular C3	42	70.0
IgG alone	16	26.7
IgG + C3	42	70.0
Weak IgM	4	6.7
Weak IgA	3	5.0
Negative DIF	2	3.3

DIF intensity and clinical severity

DIF intensity showed a statistically significant association with clinical severity. Negative staining was seen in 2 (11.1%) of 18 mild cases and was not observed in moderate or severe disease (Table 5).

**Table 5:** Association between DIF intensity and clinical severity Data are presented as number and percentage, n (%). Percentages in the mild, moderate, and severe columns were calculated within each clinical severity group. The association between DIF intensity and clinical severity was assessed using the chi-square test. A p-value of <0.05 was considered statistically significant, and p<0.001 was considered highly significant. DIF, direct immunofluorescence

DIF intensity	Mild, n=18	Moderate, n=26	Severe, n=16	Total, n=60	Test statistic	p-Value
Negative	2 (11.1%)	0 (0.0%)	0 (0.0%)	2 (3.3%)	χ² = 40.62; df = 6	<0.001
1+ weak	10 (55.6%)	4 (15.4%)	0 (0.0%)	14 (23.3%)		
2+ moderate	6 (33.3%)	16 (61.5%)	3 (18.8%)	25 (41.7%)		
3+ strong	0 (0.0%)	6 (23.1%)	13 (81.2%)	19 (31.7%)		

**Figure 1:**
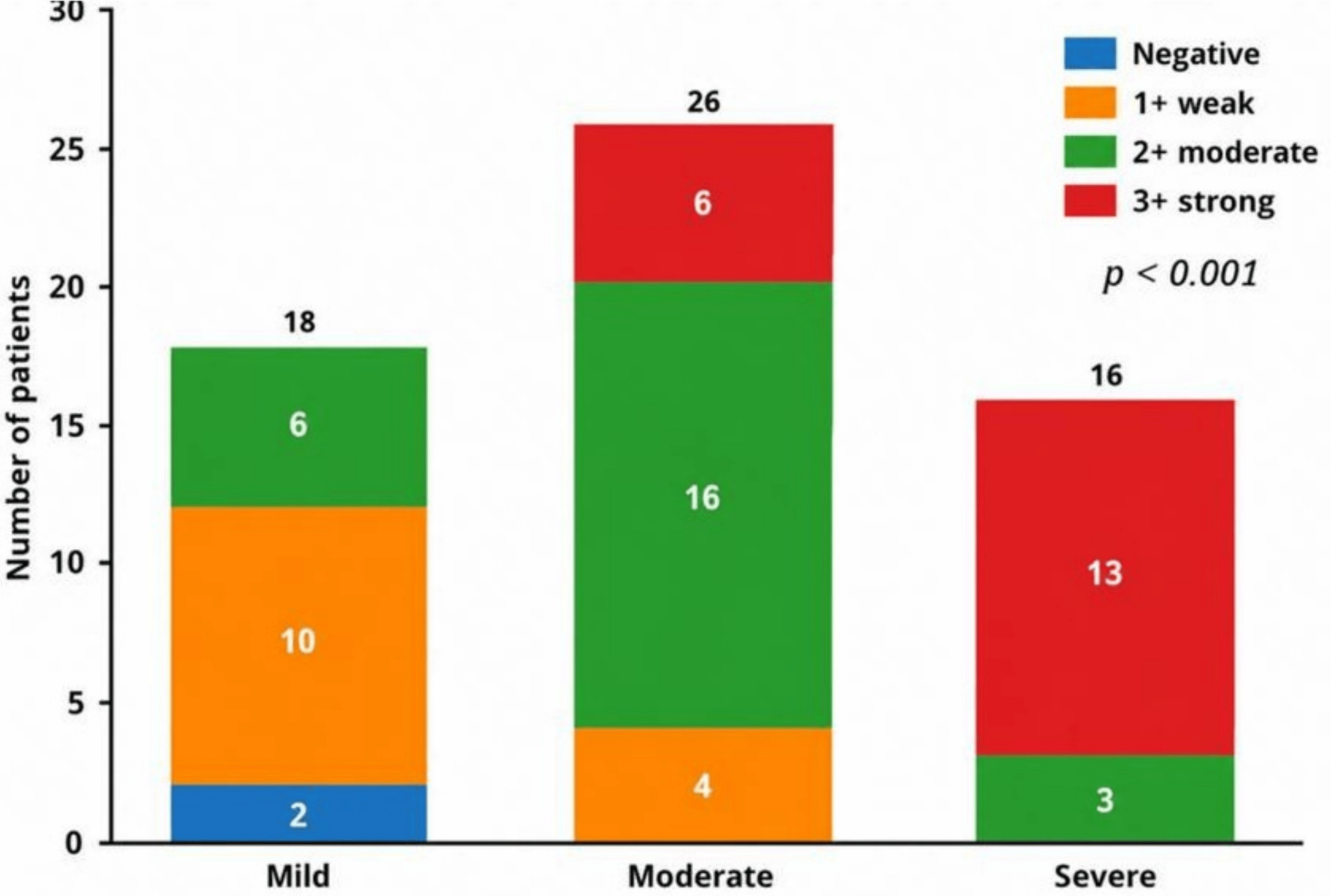
Association between DIF intensity and clinical severity Stacked bar chart showing the distribution of DIF intensity across mild, moderate, and severe disease categories. Data are presented as number of patients, n. The association was assessed using the chi-square test or Fisher’s exact test, as appropriate. A p-value of <0.05 was considered statistically significant, and p<0.001 was considered highly significant. DIF, direct immunofluorescence

Weak intensity was seen in 10 (55.6%) of 18 mild cases and 4 (15.4%) of 26 moderate cases. Moderate intensity was seen in 6 (33.3%) of 18 mild cases, 16 (61.5%) of 26 moderate cases, and 3 (18.8%) of 16 severe cases. Strong intensity was seen in 6 (23.1%) of 26 moderate cases and 13 (81.2%) of 16 severe cases. The association between DIF intensity and clinical severity was statistically significant by chi-square test, χ² = 40.62, df = 6, p<0.001 (Figure 1).

Correlation between DIF intensity and PDAI score

The mean PDAI score showed a gradual increase with rising DIF staining intensity. Patients with weak DIF positivity had a lower mean PDAI score of 11.8 ± 4.3, indicating milder clinical involvement. In comparison, patients with strong DIF intensity had a much higher mean PDAI score of 45.6 ± 12.1, reflecting more extensive disease activity (Table 6).

**Table 6:** Mean PDAI score according to DIF intensity Data are presented as number of patients, n, and mean ± standard deviation (SD). The relationship between DIF intensity and PDAI score was assessed using Spearman’s rank correlation coefficient. A p-value of <0.05 was considered statistically significant. DIF, direct immunofluorescence; PDAI, pemphigus disease area index

DIF intensity	Number of patients	Mean PDAI score ± SD
Negative	2	8.5 ± 2.1
1+ weak	14	11.8 ± 4.3
2+ moderate	25	29.7 ± 10.6
3+ strong	19	45.6 ± 12.1

**Figure 2:**
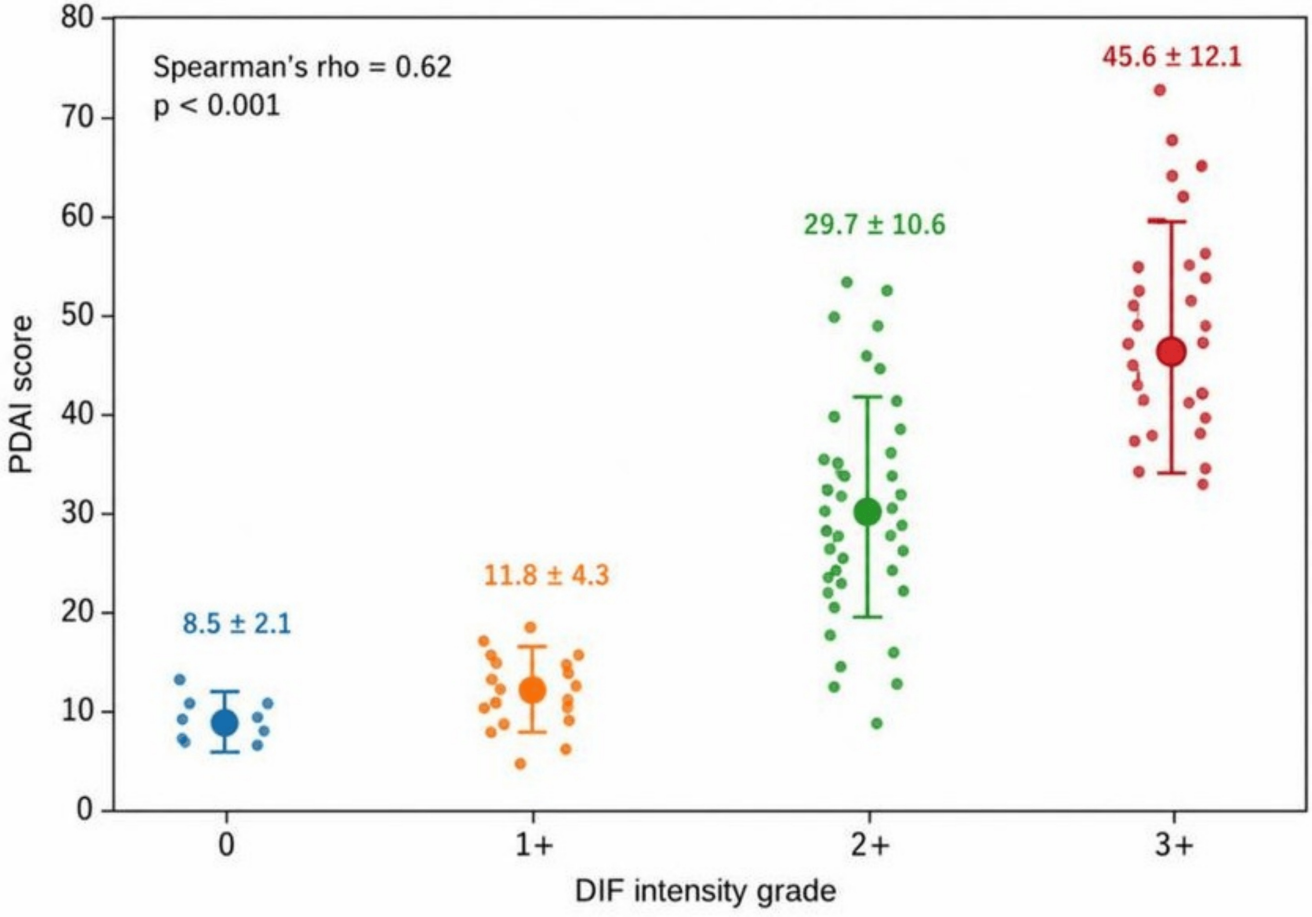
Correlation of DIF intensity with PDAI score Scatter plot showing the relationship between DIF intensity grade and PDAI score. Data are presented as individual patient values with mean ± SD. Correlation was assessed using Spearman’s rank correlation coefficient. A p-value of <0.05 was considered statistically significant, and p<0.001 was considered highly significant. DIF, direct immunofluorescence; PDAI, pemphigus disease area index

This pattern suggests that stronger tissue-bound immune staining was associated with greater clinical severity in pemphigus vulgaris (Figure 2).

Association of C3 deposition with disease severity

C3 deposition also showed a significant association with disease severity. C3 positivity was seen in 8 (44.4%) of 18 mild cases, 20 (76.9%) of 26 moderate cases, and 14 (87.5%) of 16 severe cases. When moderate and severe cases were grouped together and compared with mild cases, C3 deposition was significantly more frequent in moderate-to-severe disease, χ² = 8.00, df = 1, p = 0.004 (Table 7).

**Table 7:** Association between C3 deposition and disease severity Data are presented as number and percentage, n (%). Percentages in the mild, moderate, and severe columns were calculated within each clinical severity group. For statistical analysis, moderate and severe cases were grouped together and compared with mild cases. The association between C3 deposition and disease severity was assessed using the chi-square test. A p-value of <0.05 was considered statistically significant.

C3 deposition	Mild, n=18	Moderate, n=26	Severe, n=16	Total, n=60	Test statistic	p-Value
Present	8 (44.4%)	20 (76.9%)	14 (87.5%)	42 (70.0%)	χ² = 8.00, df = 1	0.004
Absent	10 (55.6%)	6 (23.1%)	2 (12.5%)	18 (30.0%)		

**Figure 3:**
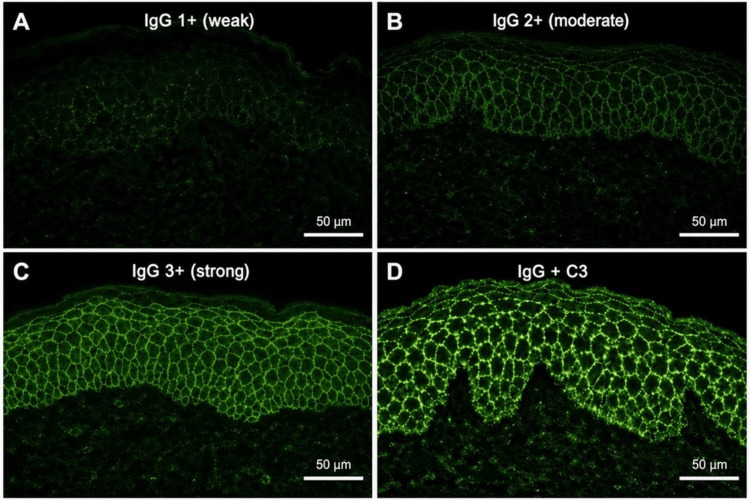
DIF dindings in pemphigus vulgaris Showing the semi-quantitative grading of intercellular IgG staining in pemphigus vulgaris. Panel A shows weak IgG staining graded as 1+, panel B shows moderate IgG staining graded as 2+, and panel C shows strong IgG staining graded as 3+. Panel D demonstrates combined intercellular IgG and C3 deposition. DIF, direct immunofluorescence

This distribution indicates that complement deposition was more frequent in patients with greater clinical disease activity. Combined intercellular IgG and C3 deposition showed a statistically significant association with moderate-to-severe pemphigus vulgaris, suggesting that the presence of C3 along with IgG may reflect a higher burden of immune-mediated tissue injury (Figure 3).

## Discussion

Pemphigus vulgaris is an autoantibody-mediated blistering disorder in which tissue injury occurs mainly at the keratinocyte surface. DIF remains a central diagnostic test because it demonstrates tissue-bound immune deposits in the affected epithelium. In the present study, DIF was positive in 96.7% of patients, and intercellular IgG deposition was the most frequent finding. This pattern is in keeping with the classical immunopathology of pemphigus vulgaris, where IgG is deposited around keratinocytes in a fishnet or chicken-wire pattern [4,5].

The demographic and clinical profile of the present cohort was also consistent with the usual presentation of pemphigus vulgaris. The mean age of the patients was 42.6 years, and there was a slight female predominance. Most patients had mucocutaneous disease, with oral erosions being the most common clinical manifestation. This is expected, as oral mucosal involvement is frequently the earliest and most persistent feature of pemphigus vulgaris and may precede skin lesions by several weeks or months [1,3].

The main observation of this study was that DIF intensity showed a moderate positive association with PDAI score. Patients with weak DIF staining were more often in the mild disease group, whereas strong intercellular IgG staining was seen more frequently among patients with higher PDAI scores and more extensive clinical disease. This finding suggests that the burden of tissue-bound immune deposition may parallel clinical activity at initial presentation. However, this association should be interpreted cautiously. The study design allows assessment of correlation at baseline, but it does not establish that DIF intensity can predict future disease course, relapse, remission, or treatment response.

C3 deposition was present in 70% of patients and was significantly more frequent in moderate-to-severe disease. This observation is biologically reasonable because complement activation may contribute to local inflammation and tissue injury in active lesions. At the same time, pemphigus vulgaris is not entirely complement-dependent. Acantholysis can occur through direct antibody-mediated disruption of desmosomal adhesion and other intracellular signalling pathways. Therefore, C3 deposition should be viewed as an additional supportive finding rather than an independent marker of disease severity [5,9].

Much of the existing literature has focused on circulating anti-desmoglein 1 and anti-desmoglein 3 antibody levels in relation to disease activity. Anti-Dsg3 antibodies are usually associated with mucosal disease, while anti-Dsg1 antibodies are more closely related to cutaneous involvement [10,11]. Serum ELISA has the advantage of being quantitative and can be useful during follow-up. However, ELISA may not be available in all clinical settings. DIF is more widely used as part of the routine diagnostic workup in dermatology and pathology laboratories. In this context, the present study adds useful information by examining whether semi-quantitative tissue-based DIF findings, especially IgG intensity and C3 deposition, are associated with PDAI-based clinical severity at presentation.

The novelty of this work lies in its focus on tissue-bound immune deposits rather than only circulating antibody titers. Previous studies have more often addressed the relationship between serum autoantibody levels and clinical severity, whereas fewer have evaluated whether DIF intensity itself has clinical relevance [10-17]. By using PDAI, a validated severity score, this study attempted to provide a structured comparison between immunopathological findings and clinical burden. The gradual increase in mean PDAI score from weak to strong DIF staining supports the possibility that detailed DIF reporting may add practical information beyond a simple positive or negative result.

The observed correlation was moderate, not absolute. This is important because the clinical expression of pemphigus vulgaris depends on several factors beyond the amount of tissue-bound IgG seen on DIF. These include antibody specificity, pathogenic potential of antibodies, epitope profile, disease duration, mucosal involvement, secondary infection, extent of erosions, and previous treatment exposure. DIF intensity itself may also vary according to biopsy site, whether the sample is taken from skin or mucosa, distance from the active lesion, transport medium, tissue preservation, processing quality, microscope settings, and observer interpretation. These factors may partly explain why DIF intensity cannot be expected to match clinical severity in every patient [5].

The possible influence of biopsy site deserves particular attention. Perilesional skin and mucosal biopsies may not always show identical staining intensity because epithelial thickness, site-specific immune deposition, and tissue handling can vary. In the present study, DIF was performed on perilesional skin or mucosal specimens according to the site of active disease and feasibility of biopsy. However, the study was not powered to perform a detailed subgroup analysis comparing skin and mucosal biopsy intensity patterns. This has been acknowledged as a limitation, and future studies should examine whether biopsy site independently affects DIF intensity and its relationship with PDAI score.

Prior treatment is another relevant confounder. Systemic corticosteroids and immunosuppressive agents may reduce immune deposition and alter DIF staining intensity. To reduce this effect, patients who had received systemic corticosteroids or immunosuppressive therapy for more than two weeks before biopsy were excluded. Even so, shorter treatment exposure, topical therapy, or variation in disease duration before biopsy may still have influenced staining intensity. Therefore, the findings should be considered in the context of baseline clinical assessment rather than as a definitive measure of disease activity.

The semi-quantitative nature of DIF grading is also an important methodological issue. In this study, DIF intensity was graded as 0, 1+, 2+, and 3+ based on the visual assessment of intercellular fluorescence. Representative images were added to illustrate weak, moderate, and strong staining patterns. This improves transparency and helps readers understand how the grading was applied. However, formal interobserver reliability testing was not performed. Observer blinding and reproducibility assessment would have strengthened the methodology. Future work should include blinded assessment by more than one dermatopathologist and calculate interobserver agreement for DIF intensity grading.

The use of PDAI strengthened the clinical assessment. PDAI provides a structured method to assess skin, scalp, and mucosal activity and is more reproducible than informal clinical judgement alone [16-20]. In the present study, PDAI helped categorize disease into mild, moderate, and severe groups and allowed comparison with DIF intensity. The progressive rise in mean PDAI score across DIF grades supports the clinical relevance of semi-quantitative DIF reporting, although it does not prove prognostic value.

From a practical perspective, DIF reports in pemphigus vulgaris may be more informative when they include the site of deposition, pattern of staining, immune reactants detected, and approximate intensity. A report describing strong intercellular IgG with C3 deposition may alert the clinician that the tissue sample shows a high burden of immune deposition. However, treatment decisions should not be based on DIF intensity alone. PDAI score, mucosal involvement, patient symptoms, comorbidities, infection risk, treatment history, and response to therapy remain central to clinical management.

Limitations

This study has several limitations. The sample size was modest, and the study was conducted at a single center, which may limit generalizability. DIF intensity was assessed semi-quantitatively, and formal interobserver reliability was not evaluated. The influence of biopsy site, disease duration, and shorter prior treatment exposure could not be fully adjusted in the analysis. Circulating anti-desmoglein 1 and 3 antibody titers were not available for all patients, and, thus, the relationship between tissue-bound immune deposits and serum autoantibody levels could not be assessed. Follow-up DIF and serial PDAI measurements were also not performed; therefore, the study cannot determine whether DIF intensity predicts treatment response, remission, or relapse.

Despite these limitations, the findings suggest that DIF may provide useful supportive information in addition to confirming the diagnosis. Stronger intercellular IgG staining and combined IgG-C3 deposition were associated with higher clinical severity at presentation. These results should be interpreted as an association rather than evidence of predictive or prognostic utility. Larger prospective studies with standardized biopsy site documentation, blinded DIF grading, interobserver reliability testing, anti-desmoglein ELISA correlation, and longitudinal follow-up are needed to clarify whether DIF intensity has a role in monitoring disease activity over time.

## Conclusions

In this study, DIF intensity was significantly associated with clinical severity in pemphigus vulgaris. Patients with stronger intercellular IgG staining and combined IgG-C3 deposition were more often found in the moderate and severe PDAI categories. These findings suggest that semi-quantitative DIF reporting may add useful supportive information at baseline, particularly when interpreted together with clinical scoring. However, DIF should still be regarded mainly as a diagnostic investigation. The present findings show an association only and do not establish DIF intensity as an independent marker for prognosis, relapse, or treatment response. Clinical assessment, PDAI scoring, biopsy site, treatment history, and patient-related factors should all be considered while interpreting DIF findings.
